# Corruption, public trust and medical autonomy in the public health sector of Montenegro: Taking stock of the COVID-19 influence

**DOI:** 10.1371/journal.pone.0274318

**Published:** 2022-09-08

**Authors:** Ivan Radević, Nikša Alfirević, Anđelko Lojpur

**Affiliations:** 1 University of Montenegro, Faculty of Economics, Podgorica, Montenegro; 2 University of Split, Faculty of Economics, Business and Tourism, Split, Croatia; Universita degli Studi di Pisa, ITALY

## Abstract

In this paper, we analyze the influence of corruption perception, experiences of corruptive behavior, and healthcare autonomy on the public trust in Montenegrin healthcare, by surveying the general population before and after the global COVID-19 pandemic. By providing a quasi-replication of a previous empirical study of corruption and trust in the Croatian public healthcare sector, we introduce the COVID-19 pandemic as a new research context. Before the pandemic, we found a consistent and significant negative influence of the corruptive practices and the generally perceived level of corruption (corruption salience) on the trust in public healthcare. The emergence of COVID-19 had mixed effects: while there is a slightly higher effect of corruption salience to the preference of public healthcare, corruptive experiences still matter but are tolerated much higher than before the pandemic. Public assessment of the autonomy of the health system increases preference for public healthcare, both before and after the pandemic, although the emergence of COVID-19 somewhat lowers this effect. The obtained results point to the most significant challenges of the ‘post-COVID-19’ social context to public health policymaking and management of public healthcare institutions. These include focusing the public healthcare reforms on corruption, reducing waiting times for different diagnostics and medical procedures in the public healthcare system, and regulating the ‘dual practice’ (simultaneous work in public and private healthcare institutions).

## Introduction

Corruption and public trust are the topics of choice when discussing the public sector and its legitimacy, as they drive the political and socioeconomic outcomes of the public sector’s functioning [1]. In the specific circumstances of the COVID-19 pandemic, emerging research confirms that the high capacity of the national state institutions, which includes the political accountability and trust of its citizens in institutions, could be beneficial in containing the worst effects [2].

However, another relevant issue has not been adequately discussed in the emerging literature: does the COVID-19 pandemic context make the citizens more tolerant of public healthcare corruption, which could lead to the severe (mis)uses of public trust under claims of addressing the crisis? Along with the lower availability of public healthcare services, as the system becomes overburdened with COVID patients, the implied and highly tolerated corruption could also lower the quality of public healthcare.

This presents a powerful argument in favor of the empirical research of corruption and social trust in the public healthcare sector and the identification of factors potentially contributing to the change of public perceptions before and after the emergence of the COVID-19 pandemic. The social dynamics of public trust matter in times of pandemic, as the higher trust in the health care system correlates with the long-term acceptance of the public health mandates, such as avoiding unnecessary travel [3]. As shown by Naher et al. [4], in their systematic review of literature related to corruption and governance of public health systems in Asia, there is a cumulative effect of corruptive activities on health outcomes, further reducing the trust in the public health system and its ability to respond to a health crisis. A link between corruption, inefficient governance of the public health systems, and inadequate responses to epidemic or pandemic outbreaks has already been detected in the case of Ebola [5]. Citizen and community outreach, directed toward a higher level of trust in the public health system, have been cited as one of the critical epidemic/pandemic responses.

It could be expected that the same applies to the case of the COVID-19 pandemic. In addition, it would be most helpful to identify the potential predictors of such patterns, which has not been done in the extant literature.

We chose to address the research question of how the perceived level of corruption (i.e., its salience), the experience of corruption, and the perception of the public healthcare system’s independence from political/government pressures (i.e., its autonomy) affect the trust in the public healthcare system. The dynamics of the distrust in the healthcare system have been linked to poor governance and politically influenced decision-making, especially related to investments in healthcare infrastructure, equipment and pharmaceutical products [4]. Therefore, assessing the public healthcare system autonomy should represent a good proxy for evaluating the healthcare policies and governance. In addition, we are interested if the emergence of COVID-19 introduces new empirical patterns into the relationship between these variables. We believe this is a worthwhile addition to the healthcare corruption literature and the body of policymaking evidence since understanding the corruption in the social landscape shaped by the pandemic proves to be very limited.

This research is significant for theory-building and relevant for policymaking and medical practice. In our research arena, located in the South East European (SEE) region, other studies have pointed to corruption as one of the primary public health concerns [6–11]. Beyond the traditional argument of structural corruption, related to the ex-communist nomenklatura [12], there are arguments based on national and legal culture, classifying only cash payments as a form of corruption and overlooking nepotism or in-kind gifts [13]. At the same time, in-kind gifts remain a common and problematic form of healthcare corruption in the region, which is not even recognized as such [14]. In this paper, we will consider similar scenarios of corruptive behavior, especially involving ‘dual practice,’ i.e., simultaneous employment in public and private sectors. In such a setting, corrupted individuals might be providing a poor service (or no service at all) in the public healthcare settings to drive the patients into the private practice [15]. The emergence of the COVID-19 pandemic calls for additional research on public health capacity and the factors contributing to it (or hindering it), especially in low- and middle-income countries [16], to avoid potential public health disasters.

In this paper, we analyzed data from two periods-before and after the emergence of COVID-19-on a nationally representative sample of adult citizens of Montenegro. We found a considerable increase in corruption salience and corruptive practices in healthcare in the post-COVID period. There are also significant differences in how medical autonomy and corruption influence the public trust in the healthcare sector before and after COVID-19. In the pre-COVID period, the conventional view of medical autonomy as increasing and the corruption as decreasing the public trust in healthcare are empirically confirmed. In the post-COVID period, the general level of perceived corruption in the public healthcare sector still reduces public trust, while the actual corruptive practices are more tolerated. The public perception of medical autonomy does not significantly influence public trust. While the doctors and the nurses could have been applauded for their contributions to public health, such public acts of support did not make any real change to the healthcare system.

The empirical results are believed to be essential for public policy and management of public healthcare in Montenegro and other developing countries, which will be further analyzed in the discussion section of this study.

## Corruption in public healthcare

In their meta-analysis of the potential policy interventions, Gaitonde et al. [17] define corruption in terms of an individual, or a group, abusing the entrusted social position or power to obtain unfair benefits, regardless of their nature (i.e., whether they are monetary, or lead to social or career advancement). According to Vian [18], it also includes fraudulent behavior (i.e., intentional rule-breaking) and abuse (i.e., circumventing the rules or taking advantage of unregulated situations). Graycar [19] warns of the ‘protean’ nature of the term, which calls for a more contextualized definition of each corruptive practice, involving identifying its type (bribery, extortion, information/resource misuse…), activity, sector, and the place of occurring.

Different stakeholders could be involved in the corruptive practices in the healthcare sector, although the generic ones include the insured citizens/patients; service providers; payers–whether public or private; regulators and other relevant government entities; and drugs/equipment providers [20]. Although corruption can occur in any of their interactions, it is most visible in direct delivery of healthcare services, i.e., direct patient contact, where informal or unjustified out-of-pocket payments could be demanded. Nevertheless, corruption can also take indirect forms, such as theft of drugs and equipment might be taking place, or the workload could be reduced by using absenteeism. In addition to asking for bribes, subtler techniques could be used, which might not even be perceived as corruptive. As previously discussed, those involve in-kind gifts, driving or forcing patients into the private practice (if the dual practice is legal), etc.

Corruption is one of the vital issues negatively impacting the global health outcomes, as it might completely discourage citizens from seeking healthcare services or lead to an informal ‘two-tiered’ system within the public healthcare sector [21], especially in the case of the ‘dual practices,’ with the less wealthy citizens being practically excluded from the provision of high-quality care. Other effects of corruption in the public sector include increased healthcare costs and higher inequality, making it challenging to introduce reforms and improve governance of the public healthcare systems [22]. Corruption also significantly decreases general satisfaction with the healthcare system, which has been empirically shown in the case of twelve post-Soviet nations by Habibov [23].

The adverse outcomes of corruption are confirmed by empirical results, linking its level to health outcomes and other quality of life indicators. In European countries, with a higher level of corrupted health systems and lower levels of income, health for citizens older than 50, in terms of chronic diseases, is significantly worsened [24]. The healthcare corruption level also proved to be a significant predictor of a variety of health indicators, including the mortality rate of children under the age of five [25], antibiotic resistance [26], COVID-19 case fatality rates during the pandemic [27], etc. Factor & Kang [28] used a dataset of 133 countries to show that higher corruption can be linked to the lower levels of national income and health costs, together with the autocratic tendencies of the government(s). The extant literature also includes an extensive discussion of the relationship between corruption and public trust, to be presented in the next section.

### Trust in public institutions, public sector corruption, and the COVID-19 pandemic

Public trust in institutions can be defined in terms of how citizens perceive social institutions’ work, compared to the widely shared normative expectations [29, 30]. It develops through communication and the cooperation of social actors included in the process to form a sort of public good [31]. As such, it is inherently linked to citizens’ evaluation of public institutions and how well those represent their interests and include them in the democratic processes [32].

As Radin [33] argued, corruption has a bi-directional causal relationship with public trust in institutions: lack of trust can fuel corruption and erode social cooperation, legitimacy of the government, and democracy [1]. This causal direction is supported by empirical evidence [34], showing that public distrust increases the perceptions of corruption (as measured by the anti-corruption policy attitudes). The resulting social capital [35], i.e., networks of trust and cooperation among corrupted public officials and associates [36], reinforce a ‘vicious circle’ of low public trust, leading to higher perceived corruption, inadequate governance, more corrupt acts and low economic development. Simultaneously, an opposite causal direction is entirely possible, as increasing corruption undermines trust in political institutions and government, leading to widespread cynicism and resentment of the general population and further inhibiting economic development [37].

Regardless of the causal direction, an association between trust in institutions and corruption is based on a strong coupling of perceptions related to the social acceptance of corruption and the legal systems’ and public institutions’ lack of legitimacy [38]. Suppose an individual cannot receive satisfactory service from a public official or an institution. In that case, they will be motivated to offer a bribe, a ‘kickback,’ or to engage in another form of corrupt behavior. Such an action will be fueled by a belief that corruption is a widely accepted form of behavior in a society, which will not be subject to severe consequences or legal prosecution. This is confirmed by behavioral research involving the analysis of descriptive norms for acts of corruption [39].

Public trust is critical in crises, such as the pandemic of the COVID-19 caused by the novel coronavirus. High levels of social cohesion, denoting a high level of trust in institutions, lead to more effective solutions to crises, including health crises. This is demonstrated by the high-trust societies reaching the inflection point of the COVID-19 infection curve more quickly than the low-trust ones [40]. High trust in institutions also leads to higher public policy acceptance. In the coronavirus case, high levels of trust give governments the legitimacy to introduce public health orders, such as social distancing and lockdowns [41]. Long-term behavioral response to the pandemic seems to be driven by the general trust in government and its institutions, comparable to the empirical results of Pak, McBryde & Adegboye [42].

In addition, in crises, including the emergence of COVID-19, public spending is heavily increased. In the case of the pandemic, it is required to procure drugs and equipment and alleviate economic consequences caused by the closures of businesses and lost jobs and incomes across multiple economic sectors [43–44]. As empirically confirmed in the case of Colombia [45], these circumstances create new opportunities for corruption, as procurement procedures are relaxed and public oversight is made increasingly difficult.

Both causal paths of the public trust-corruption relationship provide opportunities to develop negative scenarios during the pandemic crises. With a view of public trust in institutions as a cause of corruption, there could be an initial strengthening of public trust as the government takes the first steps to improve the existing situation. However, this initial impulse seems to wear off quickly if the public expectations are not met in the long term. Only consistent policy choices and government response matter to increase trust and reduce the threat of corruption during the crises [46, 47]. On the other hand, a view of corruption as a potential cause of diminishing public trust is also valid. It can be empirically confirmed in countries where the COVID-19 pandemic creates a window of opportunity for corrupt governments and elites to continue or even increase the existing corruptive practices under the pretense of responding to the emergency [48]. These empirical findings support the need to explore further the public trust–corruption relationship in different countries and contexts.

## Methods

### Research objectives and study design

This study aims to assess the empirical relationships between public trust in institutions, the autonomy of the healthcare system, and healthcare corruption—including corruption perceptions and the experience of corruptive behavior in public healthcare before and after the COVID-19 pandemic. The research design replicates a previous study on public trust and healthcare corruption in Croatia, using the elections as its social context. To achieve such an objective, we added the items used by Radin [33] and the Center for Monitoring and Research Podgoricа [49] to measure public trust in healthcare institutions and corruption in public healthcare to a study adopted by Montenegro’s Ministry of Health, to monitor and evaluate the public healthcare sector performance [50].

Following the recommendations by Bettis, Helfat & Shaver [51], our approach can be described as a quasi-replication involving two similar populations and comparable healthcare systems, sharing forty-five years of common development history [52]. As Moonesinghe et al. [53] recommended, we introduce a new social context (i.e., the COVID-19 pandemic) into the empirical replication to examine the previous research results under new circumstances.

Primary data have been collected by using a nationally representative public opinion survey on the general population of Montenegro. Although public opinion can be shaped by media and powerful social actors’ narratives [54], there are few realistic alternatives to public opinion polls regarding corruption research. Direct measurement is challenging since corruption is, in most cultures, criminal (or, at least, deviant) behavior, which motivates respondents to provide socially desirable responses and downplay its occurrence [55].

We use logistics regression for statistical analysis, following the choice of methods in the original study [33] and its efficiency in predicting the binary outcomes of various variables. In the healthcare research, some of its applications included examining out-of-pocket payments for health care and their financing in India [56], describing the general level of patient satisfaction with a healthcare system [57], with a specific reference to the patients’ familiarity with the provision of health care [58], etc. Logistics regression is, also, one of preferrable methods in analyzing public sector performance in terms of binary outcomes [59].

### Research environment

The public healthcare sector in Montenegro comprises a network of healthcare institutions matching the country’s demographic parameters. The network consists mainly of state health institutions and a few private health institutions.

The network of public (state-owned) health institutions consists of 18 health centers on the primary level. The Public Health Institute provides services of primary health protection. There are eight hospitals—providers of comprehensive healthcare services. In addition to general and special hospitals, hospitals that provide health care at the secondary and tertiary level are Codra Hospital, Tesla Medical polyclinic in Berane, and the Clinical Center of Montenegro. The Emergency Medical Service provides emergency medical care at the primary level, while the Office for Blood Transfusion covers the transfusion activities. The state-owned company, the Pharmacies of Montenegro “Montefarm,” regulates the field of medicines (and other pharmacies that have signed an agreement with the Pension and Insurance Fund). At the same time, “Rudo Montenegro” supplies medical and technical devices [50].

The system is relatively centralized: most public health institutions’ managers are appointed and dismissed by the Minister of Health (except for the directors of the Clinical Centre of Montenegro and the Institute of Public Health, which the government appoints).

Funding is entrusted to the Health Insurance Fund of Montenegro, an independent financial institution for health care funding and the exercise of other rights related to health insurance. Despite the health system relying predominantly on public health facilities, citizens would prefer the private sector if given an option. Although the market share of public health institutions is dominant, private health institutions position themselves by providing specialists’ services (28.4% of the market). The only area where private health institutions record a lower user rating compared to public health institutions is the cost of services [50].

The latest available report of the European Health Consumer Index–EHCI ranked the health system Montenegro in the twenty-third position out of thirty-five. Despite the shortcomings, the waiting time for examination in public health institutions is not excessive. As many as 82.5% of users of health institutions say their appointment with chosen physicians is made within less than a week. Moreover, citizens of Montenegro rate their general satisfaction with the health system with a high mark (6.04 on a scale of 1 to 7). However, many physicians leave the public health system, move to other countries, or get employment with privately-owned health providers. In the context of this study, it should be noted that, due to the lack of personnel and identified problems in the system (de-motivation, etc.), physicians in the public sector often work extra hours in private health institutions as a part of the ‘dual practice’ [50].

### Variables and hypotheses

Due to the quasi-replication research design, preference for the public over private healthcare service was adopted as a dependent variable from the original study conducted by Radin [33]. Her arguments for using healthcare provider preference instead of directly measuring public trust were based on the theoretical and practical interdependence of the constructs. The public trust was considered as having two dimensions: one of previous experience with healthcare providers, to be measured with patient satisfaction, and another of future expectations, to be measured with provider preference. This theoretical underpinning is supported by empirical studies, using the healthcare provider preference as a proxy of public trust. For instance, Gidman, Ward & McGregor [57] use the patients’ preference for community pharmacists versus the general practitioners to recognize the patterns of interpersonal and public trust in the healthcare sector. We do not measure patient satisfaction since no sampling frame was available to select the nationally-representative patient sample. However, we acknowledge this should be done by future research.

The described choice of measuring public trust in (healthcare) institutions is not without limitations since not everyone might not have the means to afford private healthcare services. However, we used an unambiguous wording for the survey item: ‘If I could choose, I would prefer to obtain the treatment in…’ (a private over the public healthcare facility, or vice versa). Respondents’ preference is based on the trust in the public (or private) healthcare sector, rather than the ability to pay or some other factors, limiting the actual choices of healthcare facilities in everyday life.

Several independent variables were used: salience (perception) of corruption in the public healthcare sector, demands for illicit payments (bribes), being diverted/referred to the private practice of a health provider, engagement in ‘dual practice,’ and the perception of public healthcare autonomy.

Formulations of survey items for all variables can be found in the supporting files–in their original form and English translation.

Some of those are adopted from the original study [49], which uses corruption salience and the illicit payments demands. Those variables cover perceptions and the personal experience with corruptive behavior in healthcare. This makes practical sense in the SEE social environment, where respondents are often concerned with the potential breach of confidentiality or even fear of being involved in court proceedings or criminal investigations when reporting corruption. Such circumstances can be traced back to the socialist era and ascribed to the high levels of citizens’ opportunism [60].

An additional independent variable of being diverted/referred from public to private healthcare provided was introduced to capture a practice that might not be recognized as corruptive in the current social context. The involuntary choice of private healthcare provider (i.e., coercing patients to ‘choose’ private service over the public one) has already been discussed in the introduction section, as well as reflected in our wording of the survey item: ‘Have you ever experienced a situation, in which a physician, employed at a public healthcare facility, refers you to a specific private clinic, to obtain a paid service, which should be available for free at a public facility?’. It should also be mentioned that the unrealistic waiting times for public healthcare services [61] further drive such corrupt practices.

The perception of public healthcare autonomy has been introduced to capture the public evaluation of the healthcare professionals’ skills and competencies [62], as opposed to the political appointments and adjustment of professional standards to the political requirements. There are multiple considerations of the public healthcare autonomy in the public policy context, implied by the original study [33], as informal political relationships and channels prove to block reforms and policy coordination [63]. Political actors also seem keen on avoiding the complex issue of healthcare policy and reform, although it is of critical importance for citizen perceptions and political preferences [64]. This variable also proves to be critical in the COVID-19 context of this study due to the rising role of the national state and its interventionism in suppressing the COVID-19 pandemic and the increased denialism (‘conspiracy theories’) of the pandemic reality and the public health responses. Its empirical relevance for the research of public trust during the pandemic has been shown by Saechang, Yu & Li [65]. They find the autonomy of the healthcare staff to fully mediate the relationship between public trust and COVID-19 policy compliance.

Based on the original study [33] and the previous discussion of theoretical background, we formulated the following hypotheses:

H1. Both corruption salience and experiences negatively affect trust in the public healthcare system.
H2. Perception of healthcare providers’ autonomy positively affects trust in the public healthcare system.
H3. The emergence of the COVID-19 pandemic introduces new empirical patterns into the relationship between trust and corruption in the public healthcare system.

### Population, sampling, and data collection

Two waves of data collection were performed for this study, with the same research instrument used to ensure the longitudinal comparability of empirical results. In the first round, i.e., before the COVID-19 outbreak, data were collected through personal interviewing, from May 24 to June 15, 2019, using the CAPI (*Computer-Assisted Personal Interviewing*) method. Data has been collected by trained interviewers, using the tablets for data input, allowing for simultaneous storage on a remote server and control of the process in real-time. The sampling frame was constructed using publicly available data on the number of registered voters in different districts. We used a two-stage random sampling design stratified by voting districts. The sample size equals 1,769 citizens aged 18 and over. The selection of respondents within each stratum has been performed using a random selection algorithm, with the strata size reflecting the size of the adult population of each district. The data was collected in 180 Montenegrin voting districts, thus guaranteeing national representativeness. For this sample size and the total population of Montenegro of 620,739, the confidence interval of 95%, with an incidence of 50%, equals ± 2.33%.

In the second wave of data collection, i.e., after the COVID-19 pandemic, we opted for online data collection due to health and safety concerns. The second wave was performed from April 30 to May 4, 2020, using the Survey Gizmo online system. As a sampling frame, we used the pool of 12,359 adult citizens of Montenegro who responded to the online survey. The second wave sample was selected randomly from this pool using simple random sampling, i.e., choosing a subsample of 1,769 respondents corresponding to the first wave sample. Stratification was applied to collected data in the second data collection wave by grouping the respondents most similar to the respondent grouping from the pre-COVID survey into 36 strata (groups) based on the regions (north, center, south), gender, and age. As both samples had the same number of randomly selected male and female respondents and the same number of respondents from different age groups and three Montenegrin regions, the principle of nationwide representation, according to the criteria of gender, age, and place of residence, is satisfied.

Given that the target population in both samples consists of Montenegro residents aged 18 and older and that we use the same sample size as a starting point, the estimated parameters obtained from the samples are comparable.

The study, data collection, and statistical analysis methods were reviewed and approved for compliance with ethical standards of the University of Montenegro–Faculty of Economics. The practice of faculty administration in this matter includes the review of the objectives of empirical research, the nature of data collection, and the private information collected (if any), leading to the decision to (dis)approve the planned survey and the research design. In both waves of research, participation was entirely voluntary and anonymous. Our survey(s) did not include any opportunities to identify the individual responses and link them to the respondents’ identities. Answers to demographic data, which were asked from the participants, were optional. A written introduction, uploaded on a tablet device, was shown to the participants before the beginning of interviews in the first wave of data collection. The same introduction has been placed as the first (Web) page of the Internet-based survey in the second wave of data collection. It consisted of statements related to the data collection policy, management of demographic data, and the guarantee of anonymity and the cumulative (statistical) reporting of research results.

## Results

### Summary of descriptive results

Table 1 shows the socio-demographic characteristics of the sample in the pre-Covid and post-Covid studies.

**Table 1:** Socio-demographic characteristics of the sample in pre-COVID and post-COVID surveys.

Demographic	Pre-COVID sample percentage	Post-COVID sample percentage
**Gender**		
Male	49.0	45.5
Female	51.0	54.5
**Age groups**		
18–29	22.4	26.7
30–39	18.2	20.3
40–49	17.6	16.4
50–59	17.9	18.3
60-	23.9	18.4
**Education**		
No education or primary education	14.6	1.9
High school	62.9	43.0
Professional college	10.0	19.4
Higher education	12.6	35.6
**Average income**		
No income	20.3	18.6
Less than 500 EUR	64.5	37.8
501–750 EUR	9.7	25.9
751–1000 EUR	3.7	10.1
1001–1250 EUR	0.8	3.6
1251–1500 EUR	0.4	1.8
More than 1501 EUR	0.4	2.2
**Region**		
Northern Montenegro	31.0	30.9
Central Montenegro	45.0	45.2
Southern Montenegro	24.0	24.0

Source: Research results.

Since no stratification has been applied to the education and income groups, the post-Covid survey, performed entirely online, is somewhat skewed toward the higher income brackets and highly educated individuals. This is a limitation of the research, which logically follows from the availability of Internet access and information technology skills required to fill in the Web-based questionnaire.

Descriptive statistics of the public trust and the perceived corruption in Montenegrin public healthcare are presented in Table 2.

**Table 2:** Public trust and perceived corruption in Montenegrin public healthcare in pre-COVID and post-COVID surveys.

Variable	Pre-COVID sample percentage	Post-COVID sample percentage
**Provider preference**		
Private	67.1	69.3
Public	32.9	30.7
**Perceived healthcare corruption salience**		
None	6.1	3.2
Small	20.1	13.0
Neither small nor large	23.5	16.1
High	32.4	38.8
Very high	17.9	28.9
**Illicit payments made**		
No	87.7	74.3
Yes	12.3	25.7
**Referred from public to private sector provider**		
No	73.5	49.3
Yes	26.5	50.7
**Perceived medical autonomy of the public healthcare**		
Utterly limited	23.0	30.6
Somewhat limited	19.1	13.5
Neither small nor large	38.3	23.8
Limited to a small degree	14.7	20.9
Unlimited	4.9	11.2

Source: Research results.

After the COVID-19 emergence, provider preference has changed slightly toward private healthcare facilities. The corruption salience and the illicit payments made show a higher level of perceived corruption after COVID-19. Regardless of why the service has not been performed in a public facility, referrals from the public to the private sector have risen significantly. In contrast, the perceived autonomy of public healthcare seems to be rising, as well, after COVID-19.

Interdependence of corruption salience and corruptive experiences, including diverting patients to private healthcare facilities, can be confirmed for the pre-COVID period by the simple linear correlation results, showing a modest but significant positive association (at the 0.01 level). On the other hand, medical autonomy proved to have a slight negative association with both salience and experiences of corruption, which was also significant at the 0.01 level. In other words, respondents reporting greater perceptions of corruption were also more likely to report being asked to provide illicit payments (bribes) or to state they were diverted from public to private healthcare providers. Respondents reporting higher levels of the public healthcare autonomy were, simultaneously, less likely to report both perception of corruption and the experiences of corruptive behavior. The same pattern could be observed in the post-COVID survey, although linear association strength proved somewhat higher.

### Public trust, corruption, and perceptions of medical autonomy in Montenegrin healthcare

In analogy with Radin [33], we performed the binomial logistical regression of public trust, using a range of demographic characteristics, perception of medical autonomy, and different corruption indicators: corruption salience in Model A and experience of corruptive behavior (illicit payments and diverting from public to private healthcare facilities) in Model B. For the pre-COVID survey, for both models, the difference with the constant-only model was significant at the 0.01 level, as well as all the coefficients for the predictors used. Model A correctly classified 70.7% of analyzed cases and Model B 71.6%.

A range of interesting conclusions can be drawn from the empirical results of the pre-COVID survey (see Table 3). Female respondents, compared to male ones, have the increased odds of preferring the public healthcare system, as well as the older and less wealthy respondents. At the same time, the education level produced mixed results in Models A and B.

**Table 3:** Logistics regression results for the pre-COVID survey.

		Model A–Corruption salience (pre-COVID survey)			Model B–Corruption experience (pre-COVID survey)		
		Coefficient estimate	Standard error	Odds ratio	Coefficient estimate	Standard error	Odds ratio
Predictors	Gender	.055	.007	1.057	.092	.007	1.097
	Age	.364	.003	1.440	.362	.003	1.436
	Education	.015	.004	1.015	-.115	.005	.891
	Average income	-.101	.005	.904	-.107	.005	.899
	Corruption salience in public healthcare	-.092	.003	.912			
	Illicit payments made				-.934	.014	.393
	Diverted from public to private facilities				-.516	.009	.597
	Perception of autonomy in public healthcare	.255	.003	1.291	.277	.003	1.319
	Constant	-2.587	.027	.075	-2.262	.025	.104

Source: Research results.

As expected, Model A indicates that an increase in corruption salience for one unit (with the public perception or experience of all the corruption predictors being measured by the standard five-point Likert scale) decreases the odds of preferring public healthcare by 1.096. Model B results show that providing illicit payments to healthcare staff decreases trust in public healthcare: an increase of experience related to providing illicit payments, for one unit, decreased public healthcare preference by a factor of 2.544. Experiencing diverting from public to private healthcare facilities as a result of the ‘dual practice’ system also has negative implications for public trust, although somewhat less severe than the illicit payments. Namely, for each unit of the increasing experience of this issue by the general public, public healthcare preference decreases by a factor of 1.675.

In both models, one unit of increased perception of medical autonomy by the general public (measured on a five-point Likert scale) increased public healthcare preference by 1.291 (Model A) or 1.319 (Model B).

Unlike Radin [33], for her 2007 data collection wave, we obtained consistent empirical support for hypothesis H1 under the ‘business-as-usual scenario’ (before the COVID-19 pandemic), as both corruption salience and experience proved to influence trust in the public healthcare system significantly. As expected, hypothesis H2 is supported under such a scenario since medical autonomy, conceptualized in terms of independence from political actors, supports public trust in the healthcare system.

We used the same two binary logistics regression models in the post-COVID survey (see Table 4). The difference with the constant-only model was significant at the 0.01 level and all coefficients for the predictors used. Model A correctly classified 71.6% of analyzed cases and Model B 68.1%.

**Table 4:** Logistics regression results for the post-COVID survey.

		Model A–Corruption salience (post-COVID survey)			Model B–Corruption experience (post-COVID survey)		
		Coefficient estimate	Standard error	Odds ratio	Coefficient estimate	Standard error	Odds ratio
Predictors	Gender	.121	.007	1.129	.087	.007	1.091
	Age	-.027	.002	.973	-.017	.002	.983
	Education	.100	.004	1.105	.051	.004	1.053
	Average income	.091	.003	1.095	.080	.003	1.083
	Corruption salience in public healthcare	-.308	.003	.735			
	Illicit payments made				-.507	.009	.602
	Diverted from public to private facilities				-.345	.007	.708
	Perception of autonomy in public healthcare	.118	.002	1.126	.166	.002	1.180
	Constant	-.799	.024	.450	-1.420	.022	.242

Source: Research results.

After the emergence of COVID-19, the consistent influence of all demographic variables on the public healthcare preference can be observed: female respondents, as well as those, who are younger, more educated, and wealthier, have an increased odds of preferring the public healthcare system. Patterns of corruption influence on public trust have not changed. There is ample empirical support for the influence of corruption salience on the preference of public providers, as a salience increase for one unit decreases the odds of preferring public healthcare by 1.36 (Model A). An increase in experience related to providing illicit payments, for one unit, decreased public healthcare preference by a factor of 1.66. In comparison, one unit of increased experience related to diverting from public to private healthcare facilities decreased public healthcare preference by 1.41 (Model B).

Even after the COVID pandemic, there is consistent support for hypotheses H1 and H2 since public healthcare preference is increased by 1.126 (Model A) or 1.180 (Model B) for each unit of increased perception of medical autonomy.

Comparing the empirical results before and after the emergence of the COVID-19 provides empirical support for hypothesis H3, as the corruption salience has a more significant effect than before. It is somewhat surprising that the corruption experience, both in terms of providing illicit payments and being diverted to private healthcare facilities, has lower effects on public trust than before the emergence of COVID-19. Although we have not directly measured the tolerance of corruptive behavior in the public healthcare system, the fact that people reporting their own experiences of corruption have a higher trust in the public healthcare system could be interpreted in terms of a higher tolerance of corruption.

Simultaneously, after the emergence of COVID-19, there is a lower influence of the perception of medical autonomy on the trust in the public healthcare system.

Although somewhat paradoxical, the empirical results support all three hypotheses and create an excellent background for their discussion in the next section.

## Discussion

Our research takes place in the public health system of Montenegro, which shares many similar characteristics to the other SEE public health systems.

Comparative results of research conducted in 2012 and 2019 suggest that the level of corruption in the health system of Montenegro is at a similar level in both periods. While as many as 11.5% of Montenegrin citizens claimed in 2012 that there is no corruption in health care [49], only 5% of respondents gave the same answer in 2019. On the other hand, 41.8% of respondents confirmed the presence of corruption in 2019, while in 2012, the same answer was given by 43.1% of respondents [49]. In this paper, we argue that public tolerance of healthcare corruption is increasing and that the COVID-19 pandemic is used as a window of opportunity by corrupted actors in the public healthcare sector.

Descriptive statistics show that the pandemic fuels the rise of both illicit payments and the ‘dual practice’-driven diverting of patients toward private facilities, where public healthcare employees earn personal income. In the post-COVID period, increased corruption, both in terms of its salience and the actual experiences of corruptive behavior, still decreases the public trust in the public healthcare system. Still, this effect is much lower than in the pre-COVID period. These empirical results lead to the point that the corruptive window of opportunity is created by the weakness of public healthcare systems in developing countries to simultaneously accommodate many inflowing COVID-19 patients and provide quality care to the existing patients. In such a situation, it is straightforward to divert the patients, ready to provide out-of-pocket payments for the healthcare services, from public to private healthcare facilities. In a ‘dual practice’ system, this could be perfectly legal but with disastrous effects on the public trust for patients with pre-existing medical issues. We do not consider the government response to COVID-19 in this context, where private providers could be integrated into the pandemic response to meet the limitations of the public sector. We are, instead, considering the availability of medical services to the existing patients. They seem to be denied medical service since the pandemic response overwhelms the public healthcare system. In such a situation, patients with pre-existing and acute conditions might have no choice but to provide illicit payments to public sector physicians or use the private healthcare service providers.

Our empirical results support the hypothesis of the negative relationship between corruption and trust in the public healthcare system. However, the COVID pandemic makes this effect even more prominent regarding a higher tolerance of corruptive behavior. Those results could even be interpreted in terms of citizens feeling the lack of realistic, non-corruptive choices when obtaining health services during the pandemic.

As previously mentioned, linkages between inefficient public healthcare governance and pandemic responses have already been studied [5]. However, there seems to be a lack of literature on the potential influence of the specific case of the COVID pandemic on the relationships between corruption (both perceived and experienced), perceived autonomy of the public healthcare, and the public trust. Some studies hint that the perceptions of widespread corruption and bureaucratic approach to healthcare governance led to the incompetent and non-responsive pandemic response [66]. Satisfaction of the general population with the government pandemic response has also been linked to the perceptions of corruption and public trust in institutions in middle eastern Arab countries [67]. The weakening of public trust in the pandemic context has also been commented for the case of countries where government officials and other public figures were offered the COVID-19 vaccines without regard for legal procedures and ethical regards [68]. Nevertheless, there has been no empirical research on how the pandemic context influenced the general trust in public healthcare, especially from the viewpoint of patients with pre-existing and acute medical conditions.

From this viewpoint, our empirical results could be compared to the previous research in the same geographical region conducted by Radin [33] in her 2009 data collection wave. She found that the corruption experience even supports the development of social trust in the Croatian public healthcare system, which was explained by the universal healthcare insurance coverage and the prohibitive cost of private healthcare in Croatia. In the pre-COVID scenario, it seems easier and more convenient to provide an illicit payment within the public system than to pay for private medical insurance or the private health care bills.

It could also be argued that the private healthcare facilities in the SEE region rarely provide services aimed at treating acute conditions, which was the case with the COVID-19 pandemic. They tend to focus on less risky and complex diagnostics/procedures, which require long waiting times in the public healthcare system. Therefore, some corruption in the ‘dual practice’ system could even be seen as a way of securing long-term relationships with the staff of the public healthcare system, perceived as ‘gate-keepers’ to complex, life-saving medical procedures.

## Conclusion, limitations, recommendations, and future research

This study, based on two nationally representative samples of Montenegrin citizens (before and after the COVID-19 pandemic), confirmed that both corruption salience and experiences negatively affect the trust in the public healthcare system (H1). It was also confirmed that the perception of healthcare provider autonomy positively affects public trust in the healthcare system (H2). Finally, the longitudinal nature of the study revealed that the outbreak of the COVID-19 pandemic had introduced new empirical patterns into the relationship between trust and corruption in the public healthcare system (H3).

Empirical findings suggest a significant increase in the salience of corruption in public healthcare after the COVID-19 pandemic, which also applies to the experience of corruptive practices. The percentage of the general population that prefers the public to the private health care system decreased from 32.9% to 30.7%. Our results also suggest that the corruption salience after the pandemic had a more negative impact on trust in the public health system than before the pandemic. However, after the emergence of COVID-19, the negative impact of illicit payments on public trust has been reduced, along with the negative impact of referring patients to private health facilities by medical doctors from the public health sector.

These findings lead to some recommendations for public policy and management of public healthcare institutions. Anti-corruption efforts should focus on the future reforms of public healthcare systems in Montenegro and the rest of the SEE region. Special attention must be paid to the regulation of ‘dual practice,’ which contributes to medical service availability in the context of long waiting lines for different procedures in public healthcare. Still, it raises concerns about hidden corruption, i.e., referring patients to specific private clinics and using the same medical staff, who previously made it difficult, or impossible to obtain service at a public healthcare institution. At the level of individual public healthcare institutions, the waiting times for different diagnostics and procedures should be analyzed and reduced, along with the (re)consideration of the permissions to the medical staff to engage in additional work in private clinics, as a part of the ‘dual practice’ patterns.

There are several limitations of the study. Firstly, our intention to replicate a previous study [33] indirectly influenced our choice of measuring trust in public institutions (including public healthcare). Due to other factors, including socioeconomic status, waiting times for different procedures in public healthcare, perceived quality of public vs. private healthcare, etc., a more direct measure might be preferable in future studies. In future studies, researchers might look into alternative, more focused definitions of public trust.

In addition, its results are based on data obtained from a single, small European country, with the discussion based on the previous studies in the same, culturally homogenous region. The emergence of COVID-19 could have also influenced the attitudes and perceptions of the Montenegrin general population in the ways that still need to be recognized and described. Therefore, it would be helpful to further replicate the described research design in different and culturally heterogeneous social environments and recognize fundamental mechanisms influencing the social dynamics during the COVID-19 pandemic.

## Supporting information

S1 File First round of data collection (pre-COVID). (XLSX)

S2 File Second round of data collection (post-COVID). (XLSX)

S3 File Questionnaire (original). (DOCX)

S4 File Questionnaire (translated to English). (DOCX)
