# Supplementary material for: Corruption, public trust and medical autonomy in the public health sector of Montenegro: Taking stock of the COVID-19 influence
Source: PLoS One. 2022 Sep 8;17(9):e0274318. doi: 10.1371/journal.pone.0274318 (PMC9455845; doi:10.1371/journal.pone.0274318)
Supplement: S3 File — (DOCX) [file pone.0274318.s003.docx]

**SUPPORTING INFORMATION: RESEARCH INSTRUMENT (MNE)**

1. Pol:

1. muški
2. ženski
3. Starost:
4. manje od 20 godina
5. 20–29 godina
6. 30–39 godina
7. 40–49 godina
8. 50–59 godina
9. 60 i više godine

3. Obrazovanje:

1. bez obrazovanja
2. osnovno obrazovanje
3. srednje obrazovanje
4. više obrazovanje
5. visoko obrazovanje.

4. Zanimanje:

1. đak/student
2. zaposlen u državnoj instituciji
3. zaposlen u privatnom sektoru
4. privatni preduzetnik – vlasnik
5. penzioner
6. poljoprivrednik
7. nezaposlen, traži posao
8. nezaposlen, ne traži posao
9. ostalo ______________________.

5. Koliki su Vaši prosječni mjesečni prihodi?

1. nemam prihoda (0 eura)
2. manje od 500 eura
3. 501–750 eura
4. 751–1000 eura
5. 1001–1250 eura
6. 1251–1500 eura
7. više od 1500 eura

6. U kojoj mjeri je, po Vašem mišljenju, korupcija prisutna u zdravstvenom sistemu Crne Gore?

1. nema je uopšte
2. prisutna je u maloj mjeri
3. i jeste i nije prisutna
4. prisutna je
5. prisutna je u velikoj mjeri
6. ne znam

7. Da li ste u posljednjih godinu dana Vi ili član Vaše porodice nezvanično dodatno plaćali zdravstvenom radniku u zdravstvenoj ustanovi radi obavljanja određene procedure koja je inače pokrivena zdravstvenim osiguranjem?

1. da, jedanput
2. da, više puta
3. ne
4. ne znam/ne sjećam se

8. Da li Vam se ikada desilo da Vas ljekar iz državne ustanove uputi na tačno određenu privatnu kliniku zbog usluge koju besplatno možete da dobijete u državnoj, a koju morate da platite u privatnoj klinici?

1. desilo se jedanput
2. desilo se više puta
3. nije se desilo
4. ne znam/ne sjećam se

9. Kada bih mogao da biram, radije bih se liječio:

1. u privatnoj nego u državnoj zdravstvenoj ustanovi
2. u državnoj nego u privatnoj zdravstvenoj ustanovi.

10. Crnogorski zdravstveni sistem je autonoman, tj. nije podložan uticajima političkih faktora.

1. apsolutno se ne slažem
2. djelimično se ne slažem
3. nijesam siguran
4. djelimično se slažem
5. apsolutno se slažem

11. U kojoj regiji u Crnoj Gori živite?

1. Centralna regija

2. Južna regija

3. Sjeverna regija
