# Supplementary material for: Corruption, public trust and medical autonomy in the public health sector of Montenegro: Taking stock of the COVID-19 influence
Source: PLoS One. 2022 Sep 8;17(9):e0274318. doi: 10.1371/journal.pone.0274318 (PMC9455845; doi:10.1371/journal.pone.0274318)
Supplement: S4 File — (DOCX) [file pone.0274318.s004.docx]

SUPPORTING INFORMATION: RESEARCH INSTRUMENT (ENG)

1. Gender:

1. male
2. female
3. Age
4. under 20
5. 20–29
6. 30–39
7. 40–49
8. 50–59
9. 60 and over

3. Education:

1. without education
2. primary education
3. secondary education
4. higher education
5. university education

4. Occupation:

1. pupil/student
2. civil servant
3. private sector employee
4. entrepreneur – owner
5. retiree
6. farmer
7. unemployed, looking for a job
8. unemployed, not looking for a job
9. other______________________.

5. What is your average monthly income?

1. I do not have any income (0 eur)
2. under 500 eur
3. 501–750 eur
4. 751–1000 eur
5. 1001–1250 eur
6. 1251–1500 eur
7. over 1500 eur

6. In your opinion, is there corruption in the health system of Montenegro and how much?

1. none
2. little
3. I am not sure
4. yes, there is
5. yes, there is a lot
6. I do not know

7. In the last year, have you or your family member paid extra money to a health care professional in a health care institution to do a procedure that is otherwise covered by health insurance?

1. yes, once
2. yes, several times
3. no
4. don't know/don't remember

8. Have you ever experienced a situation, in which a physician, employed at a public healthcare facility, refers you to a specific private clinic, to obtain a paid service, which should be available for free at a public facility?

1. it happened once
2. it happened several times
3. it didn't happen
4. don't know/don't remember

9. If I could choose, I would prefer to obtain the treatment in…:

1. in a private clinic than in a state clinic
2. in a state clinic than in a private clinic.

10. Montenegrin health system is autonomous, i.e. not subject to political factors.

1. I absolutely disagree
2. partially disagree
3. I'm not sure
4. partially agree
5. I absolutely agree

11. In which region of Montenegro do you live?

1. Central Region

2. Southern Region

3. Northern Region
